# Clinical profile of headache attributed to anxiety and depressive disorders: an observational study

**DOI:** 10.3389/fneur.2026.1805261

**Published:** 2026-05-18

**Authors:** Boyan Chen, Sailucao Zhang, Jing Yang, Xinyu Yan, Chunyu Wang, Dongjun Wan

**Affiliations:** 1 Department of Neurology, 940th Hospital of Joint Logistics Support Force of PLA, Lanzhou, China; 2 Department of Endocrinology, 940th Hospital of Joint Logistics Support Force of PLA, Lanzhou, China; 3 First Clinical College of Gansu University of Chinese Medicine, Lanzhou, China

**Keywords:** anxiety, clinical phenotype, depression, headache attributed to anxiety and depressive disorders, secondary headache

## Abstract

**Background:**

Headache attributed to anxiety or depressive disorders was added to the appendix of the International Classification of Headache Disorders, 3rd edition (ICHD-3) in 2018. However, its specific clinical features have not yet been systematically characterized, limiting its early recognition and optimal clinical management.

**Methods:**

We conducted an observational study between March 2024 and June 2025 at a headache center in China. Following stringent exclusion of primary headache disorders and other secondary headache causes, 101 patients meeting diagnostic criteria for headache attributed to anxiety and depressive disorders (HA-ADD) were enrolled. Demographic data, detailed headache characteristics, and psychiatric symptoms were collected through structured face-to-face interviews.

**Results:**

The cohort was predominantly female (70.3%; *n* = 71) with a median age of 36 years. The characteristic HA-ADD phenotype was defined by: (1) pain characteristics: bilateral pain (72.3%) with dull quality (92.1%) and moderate-to-severe intensity (53.5% moderate; 42.6% severe); (2) location: temporal (47.5%) and parietal regions (35.6%); (3) temporal pattern: 60.4% of attacks lasted less than 4 h; (4) associated symptoms: phonophobia (79.2%), restlessness/agitation (58.4%), dizziness (57.4%), and nausea (51.5%); (5) trigger factors: emotional fluctuations (71.3%) and poor sleep quality (57.4%) were predominant. Notably, 73.2% of patients experienced severe headache-related functional impairment (HIT-6 score >60).

**Conclusion:**

Headache attributed to anxiety and depressive disorders presents a distinct clinical profile characterized by bilateral, dull, moderate-to-severe pain with short attack duration and prominent associated symptoms including phonophobia and restlessness. This phenotypic signature differentiates HA-ADD from common primary headache disorders and provides clinical markers that may facilitate earlier diagnosis and appropriate therapeutic intervention.

## 1 Introduction

Anxiety and depression are closely associated with headache disorders, not only frequently co-occurring with migraines and tension-type headaches but also serving as independent causes of headache (1, 2). Although headache attributed to anxiety or depressive disorders was added to the appendix of the International Classification of Headache Disorders, 3rd edition (ICHD-3) in 2018, its clinical features have not yet been systematically characterized. This knowledge gap hinders accurate early recognition and leading to delayed diagnosis and suboptimal treatment.

A key diagnostic issue is determining whether the headache is a secondary headache attributable to anxiety and depressive disorders (HA-ADD) or a primary headache comorbid with anxiety and depressive disorders. According to ICHD-3, headache attributed to depressive disorder (code A12.3) and headache attributed to generalized anxiety disorder (code A12.8) share the same diagnostic structure: (1) a diagnosis of depressive disorder or generalized anxiety disorder meeting DSM-5 criteria; (2) headache occurs exclusively during periods of anxiety or depressive disorder; and (3) headache is not better accounted for by another ICHD-3 diagnosis. The crucial distinction lies in the presence of a temporal causal relationship. In practice, patients often struggle to accurately recall and describe the temporal relationship between the onset and fluctuations of their anxiety and depressive symptoms and their headache episodes. This substantially limits the clinical applicability of this core diagnostic criterion. Consequently, the current diagnosis of HA-ADD largely relies on a “therapeutic diagnosis” or “*post hoc* verification” approach, meaning that when the mood disorder is effectively treated and headache symptoms subsequently improve or resolve, the headache is then considered attributable to anxiety and depressive disorders.

Anxiety and depression frequently co-occur. A cohort study revealed that 67% of patients with depression had comorbid anxiety and 63% of those with anxiety had comorbid depression (3). These findings suggest that the concurrent presence of both disorders is more common than the presence of either disorder occurring in isolation. Epidemiological data indicate that major depressive disorder (MDD) and persistent depressive disorder (PDD) are the most prevalent forms of depressive disorders (4, 5). Generalized anxiety disorder (GAD) is a highly chronic condition that is independent of specific triggers. Moreover, patients with GAD and MDD exhibit particularly high rates of comorbidity. A large-scale epidemiological survey revealed that up to 65% of individuals with GAD concurrently meet the criteria for MDD (6).

Given this strong bidirectional relationship between anxiety and depression and their frequent co-occurrence, the present study focused on patients with GAD comorbid with MDD or PDD to characterize the clinical features of HA-ADD.

## 2 Patients and methods

### 2.1 Patients

This study was conducted at the Headache Center of the 940th Hospital, Joint Logistics Support Force of the PLA. Patients provisionally diagnosed with HA-ADD between March 2024 and June 2025 were enrolled. Ethical approval was obtained from the Hospital Ethics Committee (Approval No. [2024KYLL198]), and all patients provided written informed consent prior to participation.

Inclusion criteria were as follows: (i) aged 18–65 years; (ii) met the Diagnostic and Statistical Manual of Mental Disorders, 5th edition (DSM-5) criteria for MDD or PDD (F32.x/F33.x, F34.1) co-occurring with GAD (F41.1), with concomitant headache symptoms; (iii) headache occurring after anxiety and depression; and (iv) were free of antidepressant or anxiolytic medications for at least 1 month prior to enrollment (either treatment-naïve or voluntarily discontinued ≥1 month prior).

Exclusion criteria included: (i) a history of any diagnosis meeting the ICHD-3 criteria for primary headache disorders (e.g., migraine and tension-type headache); (ii) headaches attributed to other underlying causes; (iii) a diagnosis of other primary or secondary psychiatric disorders per the DSM-5 criteria (e.g., substance use disorders and psychiatric conditions secondary to medical conditions); (iv) absence of significant headache improvement after 6 months of adequate antidepressant/anxiolytic therapy; (v) pregnancy or lactation; and (vi) severe systemic disease.

### 2.2 Procedures

During the initial consultation, a headache specialist performed detailed clinical interviews and physical examinations. Demographic and headache characteristics, including age, sex, onset time, pain location, quality, intensity, duration, monthly frequency, triggers, relieving factors, associated symptoms, and current medications, were recorded. Features of mood disorders, including core symptoms and severity, were also documented. All patients completed a series of standardized assessments to quantify mood symptoms and headache impact. The final diagnosis of HA-ADD was established through a multidisciplinary discussion between at least one dedicated headache specialist and one psychiatrist, according to the DSM-5 criteria for anxiety and depression and the ICHD-3 criteria for headache disorders. To exclude secondary causes, all patients underwent appropriate ancillary investigations, including brain CT/MRI and blood tests (liver and renal function, autoimmune antibodies, erythrocyte sedimentation rate, D-dimer, etc.).

**Figure 1:**
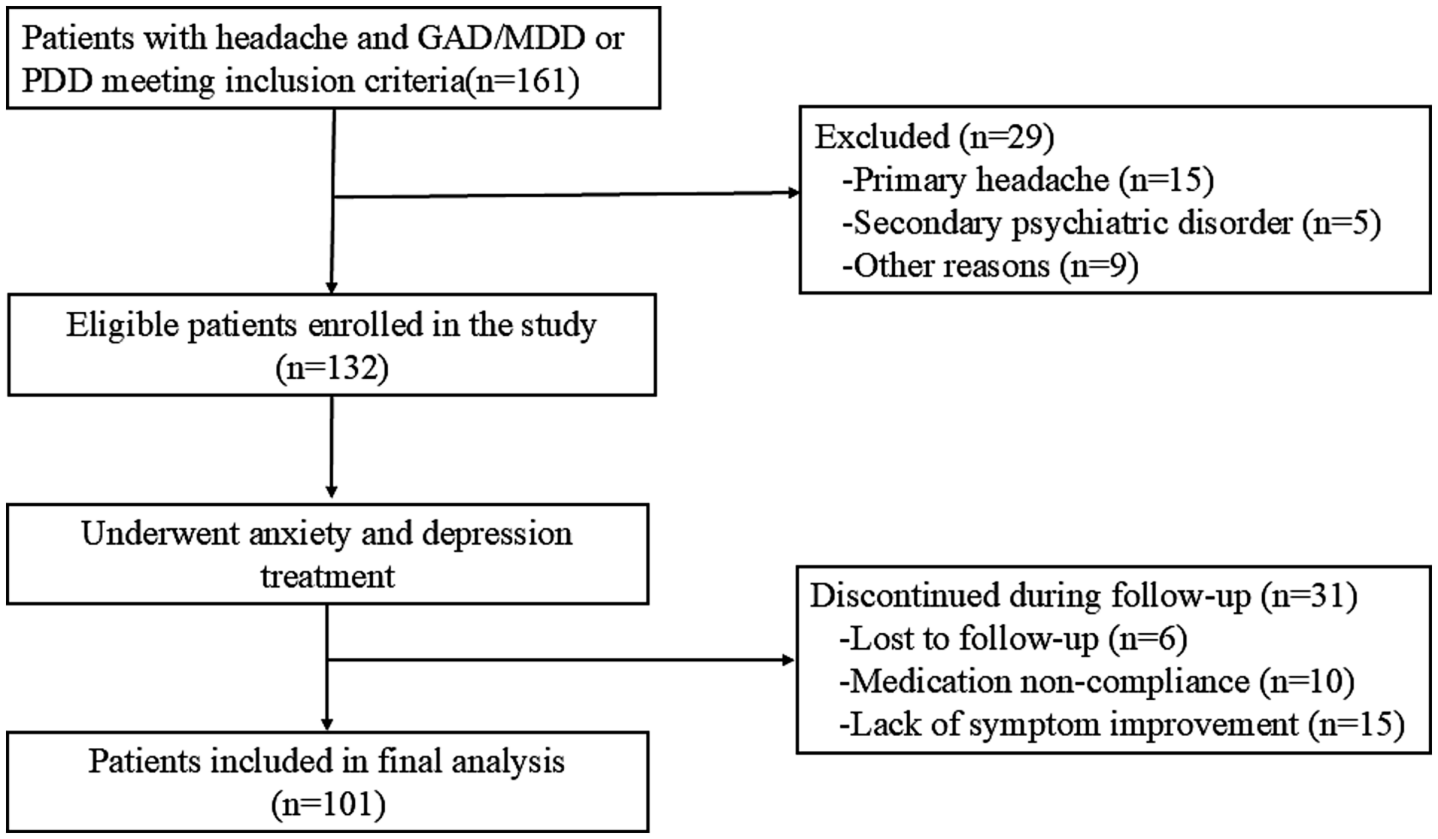
Flowchart of patient selection and outcomes. GAD, Generalized anxiety disorder; MDD, Major depressive disorder; PDD, Persistent depressive disorder.

Patients with a provisional diagnosis of HA-ADD were administered with antidepressant and anxiolytic medications. Follow-up assessments were conducted at 1, 3, and 6 months after treatment initiation, either in the outpatient clinic or via telephone, to evaluate changes in mood disorders and headache symptoms. Headache was considered attributable to anxiety and depressive disorders if it was resolved or markedly improved (in terms of frequency, severity, or duration) following the alleviation of mood symptoms. Operationally, ‘markedly improved’ was defined as at least a one-category reduction in HIT-6 severity grade (e.g., from ‘very severe impact’ to ‘substantial impact’). The detailed flowchart of patient screening and enrollment is presented in Figure 1.

### 2.3 Scale assessment

All participants were assessed using the standardized scales. Scale data were entered by one investigator and independently double-checked by another. These assessments included the following: (i) The visual analog scale (VAS) was used to assess the maximum pain intensity (0–3: mild; 4–6: moderate; 7–10: severe). (ii) The short-form Headache Impact Test-6 (HIT-6) was employed to evaluate the impact of headache on daily life and work (7). Total scores were interpreted as follows: 36–49 (little or no impact), 50–55 (moderate impact), 56–59 (substantial impact), and 60–78 (very severe impact). (iii) The Patient Health Questionnaire 9 (PHQ-9) was administered to assess the severity of depressive symptoms (8). Scores were classified as follows: 0–4 (minimal or none), 5–9 (mild), 10–14 (moderate), and 15–27 (severe). (iv) The Generalized Anxiety Disorder 7-item (GAD-7) scale was used to assess the severity of anxiety symptoms (9). Scores were categorized as follows: 0–4 (minimal or none), 5–9 (mild), 10–14 (moderate), and 15–21 (severe).

### 2.4 Treatment protocol

Patients with a provisional diagnosis of HA-ADD received individualized pharmacotherapy prescribed by a psychiatrist to target their anxiety and depressive symptoms. The treatment goal was to reach and maintain each medication within its recommended effective dose range for at least 4–6 weeks to ensure adequate efficacy, with subsequent adjustments based on individual response and tolerability. Pharmacological regimens included selective serotonin reuptake inhibitors (SSRIs; e.g., fluvoxamine maleate and escitalopram), non-benzodiazepine anxiolytics (e.g., buspirone and tandospirone), serotonin and norepinephrine reuptake inhibitors (SNRIs; e.g., duloxetine), and Chinese herbal formulations (e.g., Shuganjieyu capsule and Wuling capsule). Notably, venlafaxine and amitriptyline, both explicitly recommended in the current guidelines for migraine and tension-type headache (TTH) prophylaxis (10, 11), were excluded from this study to avoid potential confounding effects. Throughout the 6-month treatment and follow-up period, patients were permitted to use acute medications (such as nonsteroidal anti-inflammatory drugs or triptans) as needed for headache relief. Acute medications response was not systematically evaluated. No other headache preventive medications were administered during the study, including but not limited to antiepileptic drugs (e.g., topiramate, valproate, gabapentin), beta-blockers (e.g., propranolol, metoprolol), tricyclic antidepressants (e.g., amitriptyline), calcium channel blockers (e.g., flunarizine), and onabotulinumtoxin A.

### 2.5 Outcome assessment

Improvement in headache symptoms were evaluated in the follow-up assessments using three standardized instruments: the PHQ-9 for depression, the GAD-7 for anxiety, and the HIT-6 for headache impact. Clinical improvement was defined as a reduction in total scores across all three scales, with each score decreasing by at least one severity category (e.g., PHQ-9 score from severe to moderate; HIT-6 score from “very severe impact” to “substantial impact”).

### 2.6 Statistical analysis

The normality of continuous variables was assessed using the Shapiro–Wilk test. Non-normally distributed variables are consequently reported as medians and interquartile ranges (IQRs). Categorical variables are presented as frequencies and percentages. For group comparisons, the Mann–Whitney U test was used for continuous variables, and the chi–square test or Fisher’s exact test was used for categorical variables. The association between anxiety and depression was evaluated using Spearman’s rank correlation. Furthermore, linear, logistic, and negative binomial regression models were used to examine the independent effects of anxiety and depression on headache outcomes. All analyses were performed in SPSS 25.0, with a two-sided *p* < 0.05 considered statistically significant. This study followed the Strengthening the Reporting of Observational Studies in Epidemiology (STROBE) guidelines (12).

## 3 Results

Among the 132 Han Chinese patients initially enrolled with provisional HA-ADD, all received a full course of adequately dosed antidepressant/anxiolytic therapy. During the 6-month follow-up period, 6 patients were lost to follow-up, 10 were excluded for medication noncompliance, and 15 were excluded due to a lack of headache improvement. Thus, 101 patients were included in the final analysis. This cohort was predominantly female (70.3% female vs. 29.7% male), with a median age of 36 years (IQR: 25.5–48.5). Notably, the age distribution was skewed toward younger individuals, with the 18–27-year age group representing the largest proportion (37.6%), followed by a secondary peak in the 38–47-year age group (22.8%). Overall, 46.5, 26.7, and 26.7% of patients exhibited severe, moderate, and mild anxiety, respectively; the corresponding rates for depression were 64.3, 23.8, and 11.9%, respectively. No significant sex differences were observed in symptom severity (Table 1).

**Table 1:** Demographics of patients with HA-ADD.

Demographics	Male (*n* = 30)	Female (*n* = 71)	Total (*n* = 101)	*p* value
Age, Median (IQR)	27 (25–47.5)	40 (27–49)	36 (25.5–48.5)	0.274
Age distribution, *n* (%)				0.245
18–27y	16(53.3)	22(31.0)	38 (37.6)	
28–37y	3(10.0)	11(15.5)	14 (13.9)	
38–47y	4(13.3)	19(26.8)	23 (22.8)	
48–57y	4(13.3)	14(19.7)	18 (17.8)	
58–65y	3(10.0)	5(7.0)	8 (7.9)	
Education level, *n* (%)				0.238
≤Primary	4(13.3)	19(26.8)	23 (22.8)	
Secondary	12(40.0)	19(26.8)	31 (30.7)	
≥College	14(46.7)	33(46.4)	47 (46.5)	
Headache onset after anxiety/depression, *n* (%)				0.113
≤6 months	15 (50.0)	39 (54.9)	54 (53.4)	
6–12 months	5 (16.7)	2 (2.8)	7 (7.0)	
1–2 year	4 (13.3)	10 (14.1)	14 (13.9)	
≥2 year	6 (20.0)	20 (28.2)	26 (25.7)	
Anxiety, *n* (%)				0.415
Mild	9 (30.0)	18 (25.3)	27 (26.7)	
Moderate	10 (33.3)	17 (24.0)	27 (26.7)	
Severe	11 (36.7)	36 (50.7)	47 (46.5)	
Depression, n (%)				0.134
Mild	2 (6.7)	10 (14.1)	12 (11.9)	
Moderate	11 (36.7)	13 (18.3)	24 (23.8)	
Severe	17 (56.6)	48 (67.6)	65 (64.3)	

HA-ADD: Headache attributed to anxiety and depressive disorders; IQR: interquartile range.

### 3.1 Clinical features of headache

Among the 101 patients with HA-ADD, 54 (53.5%) reported moderate headache and 43 (42.6%) reported severe headache intensity. The headaches experienced by most participants were bilateral (73 cases, 72.3%), with a sex difference in pain side distribution (80.3% in females vs. 53.3% in males*; p* = 0.006). The most frequent headache locations were the temporal (47.5%), parietal (35.6%), occipital (29.7%), and frontal (26.7%) regions, followed by the entire head (7.9%). In terms of pain quality, dull pain (92.1%) was the predominant type of headache, followed by stabbing (17.8%) and throbbing (9.9%). The incidence of dull pain was significantly greater in females than in males (95.8% vs. 83.3%; *p* = 0.048) (Table 2).

**Table 2:** Clinical features of patients with HA-ADD.

Features	Male (*n* = 30)	Female (*n* = 71)	Total (*n* = 101)	*p* value
Pain side, *n* (%)				
Bilateral	16 (53.3)	57 (80.3)	73 (72.3)	0.006*
Predominant right-side	6 (20.0)	6 (8.5)	12 (11.9)	0.193
Predominant left-side	6 (20.0)	6 (8.5)	12 (11.9)	0.193
Side-alternating	2 (6.7)	2 (2.8)	4 (4.0)	0.728
Locations, *n* (%)				
Temporal	11 (36.7)	37 (52.1)	48 (47.5)	0.156.
Parietal	7 (23.3)	29 (40.8)	36 (35.6)	0.093
Occipital	9 (30.0)	21 (29.6)	30 (29.7)	0.966
Forehead	6 (20.0)	21 (29.6)	27 (26.7)	0.320
Generalized	3 (10.0)	5 (7.0)	8 (7.9)	0.921
Quality, *n* (%)				
Dull	25 (83.3)	68 (95.8)	93 (92.1)	0.048*
Stabbing	7 (23.3)	11 (15.5)	18 (17.8)	0.347
Pulling/tugging	3 (10.0)	0 (0.0)	3 (3.0)	0.024*
Throbbing	2 (6.7)	8 (11.3)	10 (9.9)	0.732
Unclassifiable	2 (6.7)	5 (7.0)	7 (6.9)	1.0
Electric shock-like	1 (3.3)	1 (1.4)	2 (2.0)	0.508
Pressing	0 (0.0)	1 (1.4)	1 (1.0)	1.0
VAS, *n* (%)				0.218
Mild	1 (3.3)	3 (4.2)	4 (3.9)	
Moderate	20 (66.7)	34 (47.9)	54 (53.5)	
Severe	9 (30.0)	34 (47.9)	43 (42.6)	
Headache Impact (HIT-6), *n* (%)				<0.001*
No/little	0 (0.0)	2 (2.8)	2 (2.0)	
Moderate	7 (23.3)	2 (2.8)	9 (9.0)	
Substantial	10 (33.3)	6 (8.5)	16 (15.8)	
Severe	13 (43.3)	61 (85.9)	74 (73.2)	

*p < 0.05; HA-ADD: Headache attributed to anxiety and depressive disorder.

**Figure 2:**
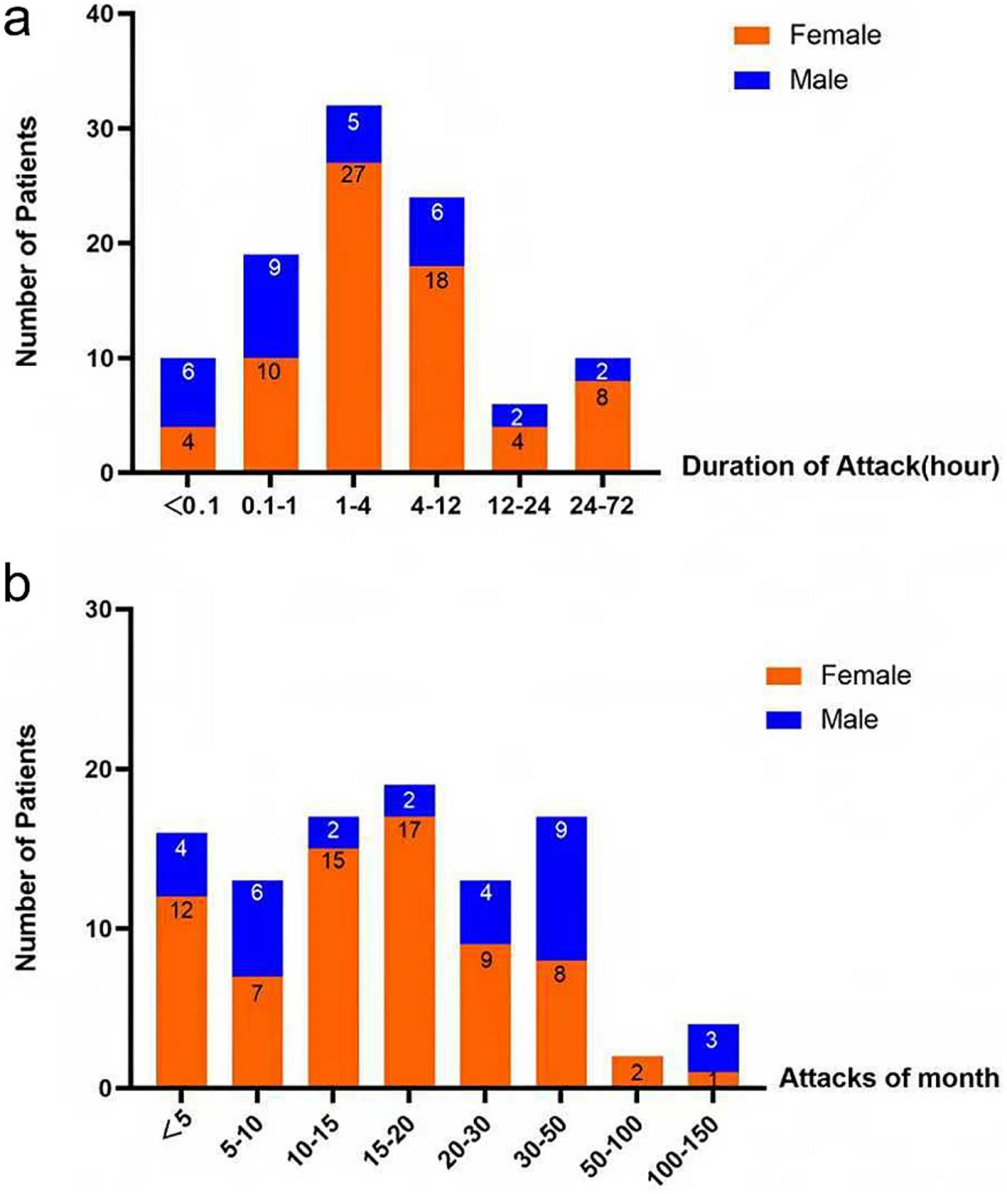
Headache attack duration and frequency in patients with HA-ADD. **(a)** Distribution of headache attack duration. Most patients experienced attacks lasting less than 4 h. **(b)** Distribution of headache attack frequency per month. Most patients experienced fewer than 20 attacks per month.

As illustrated in Figure 2a, most patients experienced relatively short headache durations. A total of 60.4% of patients reported headache episodes lasting less than 4 h, 23.8% lasting 4–12 h, 5.9% lasting 12–24 h, and 9.9% lasting 24–72 h. Concerning headache frequency, 28.7% of patients experienced fewer than 10 attacks per month, whereas 35.6% reported 10–20 attacks per month (Figure 2b). More than 60% of patients with HA-ADD experienced fewer than 20 episodes per month. Overall, differences in headache attack duration (*p* = 0.047) and monthly attack frequency (*p* = 0.015) were observed between sexes. However, following Bonferroni correction for multiple comparisons, none of the intergroup differences remained statistically significant.

Importantly, headache-related impacts on daily life were highly prevalent in the study population, with 73.2% of patients experiencing a “severe” impact (HIT-6 > 60). This impairment had a more significant effect in female patients than in male patients (*p<*0.001).

### 3.2 Associated symptoms

**Figure 3:**
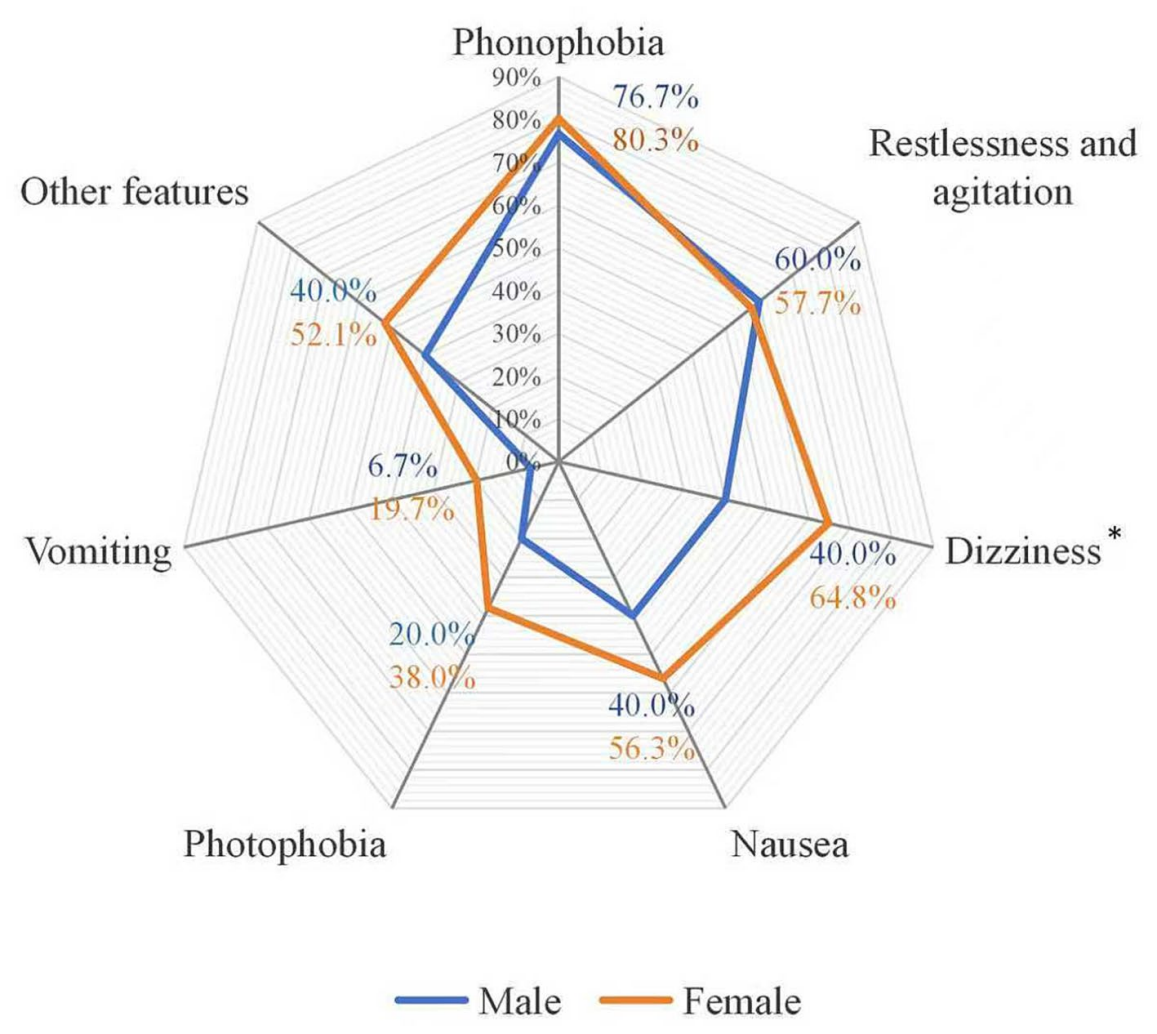
Associated symptoms in patients with HA-ADD. *p < 0.05. HA-ADD: Headache attributed to anxiety and depressive disorders.

Among the 101 HA-ADD patients, the most commonly associated symptoms were phonophobia (79.2%), restlessness/agitation (58.4%), dizziness (57.4%), and nausea (51.5%). Less frequent symptoms included photophobia (34.7%), vomiting (15.8%), and other manifestations such as sweating, lacrimation, and bitter taste (Figure 3). Dizziness was significantly more common among female patients than among male patients (64.8% vs. 40.0%; *p* = 0.001).

### 3.3 Triggers and relieving factors

The most commonly reported triggers were emotional fluctuations (71.3%), poor sleep quality (57.4%), and irritability (56.4%). Significant sex differences were observed for emotional fluctuations (77.5% in females vs. 56.7% in males; *p* = 0.035) and irritability (63.4% vs. 40.0%; *p* = 0.015). The most effective relieving factors were adequate sleep (39.6%) and lying down (38.6%). Compared with males, females reported massage as significantly more effective (21.2% vs. 0.0%; *p* = 0.006) (Table 3).

**Table 3:** Trigger and relieving factors of HA-ADD.

Factors	Male (*n* = 30)	Female (*n* = 71)	Total (*n* = 101)	*p* value
Trigger factors, *n* (%)				
Emotional fluctuations	17 (56.7)	55 (77.5)	72 (71.3)	0.035*
Poor sleep quality	14 (46.7)	44 (62.0)	58 (57.4)	0.155
Irritability	12 (40.0)	45 (63.4)	57 (56.4)	0.030*
Stress	6 (20.0)	22 (31.0)	28 (27.7)	0.259
Fatigue	5 (16.7)	20 (28.1)	25 (24.8)	0.221
Exposure to cold	3 (10.0)	12 (16.9)	15 (17.4)	0.559
Physical activity	2 (6.7)	9 (12.7)	11 (10.9)	0.592
Exposure to heat	2 (6.7)	8 (11.2)	10 (9.9)	0.732
None	5 (16.7)	4 (5.6)	9 (8.9)	0.163
Altered sleep–wake cycle	2 (6.7)	3 (4.2)	5 (5.0)	0.988
Weather changes	2 (6.7)	1 (1.4)	3 (3.0)	0.210
Menstruation	0 (0.0)	4 (5.6)	4 (4.0)	0.442
Excessive work	1 (3.3)	0 (0.0)	1 (1.0)	0.297
Relieving factors, *n* (%)				
Adequate sleep	9 (30.0)	31 (43.7)	40 (39.6)	0.200
Lying down	8 (26.7)	31 (43.7)	39 (38.6)	0.109
Quiet environment	8 (26.7)	22 (31.0)	30 (29.7)	0.664
Outdoor walking	4 (13.3)	12 (17.0)	16 (15.8)	0.880
Massage therapy	0 (0.0)	15 (21.2)	15 (14.9)	0.015*
None	3 (10.0)	9 (12.7)	12(11.9)	1.0
Removal from stimulating environments	2 (6.7)	5 (7.0)	7 (7.0)	1.0
Shifting attention	1 (3.3)	2 (2.8)	3 (3.0)	1.0
Standing	1 (3.3)	1 (1.4)	2 (2.0)	0.508
Food intake	1 (3.3)	0 (0.0)	1 (1.0)	0.297

*p < 0.05; HA-ADD, Headache attributed to anxiety and depressive disorders.

### 3.4 Associations between anxiety and depression severity and headache features

To assess the associations between anxiety and depression severity and headache characteristics, separate regression models were constructed for headache intensity (VAS), headache impact (HIT-6), attack duration, and frequency. Neither anxiety nor depression severity was related to attack duration or pain intensity. In contrast, both were significantly associated with a greater impact of headaches on normal daily activity. Furthermore, moderate depression specifically increased the risk of headache attacks (RR = 2.66; *p* = 0.007).

## 4 Discussion

In clinical practice, HA-ADD is rarely diagnosed. This underdiagnosis stems primarily from the limited understanding of its clinical characteristics and the challenges in establishing a causal relationship between headache and anxiety or depressive disorders according to the ICHD-3 diagnostic criteria. To verify that patients’ headaches were attributable to anxiety and depression, we administered adequate pharmacological treatment for these mood disorders and conducted systematic follow-up assessments. Headaches were considered secondary to the underlying affective disorders when their severity decreased along with the improvement in mood symptoms.

We identified a distinct clinical profile of HA-ADD, characterized by bilateral, dull pain predominantly localized in the temporal and parietal regions, of moderate to severe intensity, attack duration typically under 4 h, and frequent accompanying symptoms such as phonophobia, dizziness, and restlessness. This distinct profile differentiates patients with HA-ADD from those with common primary headache disorders and provides a valuable basis for earlier clinical identification of HA-ADD.

The main clinical features of HA-ADD contrast with those of migraine and TTH. While migraine is typically characterized as unilateral (56%), with throbbing (47%) or pressing quality (42%) (13), and TTH is predominantly bilateral (90%), with a pressing or tightening sensation (78%) and generally mild to moderate intensity (99%) (14), HA-ADD exhibits a consistent pattern of bilateral, dull, moderate-to-severe pain. In addition to pain characteristics, the associated symptoms offer further diagnostic clues. Patients with HA-ADD frequently reported phonophobia (79.2%) and restlessness/agitation (58%), which are symptoms that align more closely with somatic manifestations of anxiety than with typical TTH profiles (15). In contrast, migraine is commonly associated with nausea (86.1%), phonophobia (69.5%), photophobia (61.0%), and vomiting (59.2%) (16). The attack duration further distinguishes between these disorders. Most HA-ADD episodes (60.4%) were brief (<4 h), in contrast to the typical 4–72 h duration of untreated migraine and the 30-min to 7-day range of TTH (17). The pattern of frequent, short-duration attacks is a key temporal characteristic of HA-ADD. Trigger and relief factors also provide differential insights. Emotional fluctuations (71.3%), irritability (56.4%), and poor sleep quality (57.4%) were the core triggering factors for HA-ADD. In contrast, stress and sleep disturbances are well-established triggers of migraines and TTH (15, 16, 18, 19). A comparative summary of the clinical features is presented in Table 4.

**Table 4:** Comparison of clinical features of HA-ADD, migraine, and TTH.

Features	HA-ADD	Migraine	Tension-type headache
Pain type	Dull	Throbbing	Pressing/tightening
Location	Bilateral (temporoparietal)	Often unilateral	Bilateral (frontal/temporal/occipital)
Severity	Moderate to severe	Moderate to severe	Mild to moderate
Duration	Predominantly <4 h	4–72 h	30 min–7 d
Associated symptoms	Phonophobia, dizziness, restlessness/agitation	Vomiting, nausea, photophobia, phonophobia.	No more than one of photophobia or phonophobia

HA-ADD: Headache attributed to anxiety and depressive disorders; TTH: tension-type headache.

In this study, we observed that HA-ADD share several clinical features with migraine, including a higher prevalence among women, moderate-to-severe pain intensity, and accompanying symptoms such as nausea and phonophobia. These overlapping features suggest that potential shared biological mechanisms between HA-ADD and migraines. A large-scale population-based longitudinal study revealed a bidirectional association between anxiety and depression and migraine (1): anxiety and depression increase the risk of migraines (RR 1.8–2.0), and conversely, the risk of having anxiety and depression is increased among individuals with migraine (RR 1.3–1.6). Several shared pathophysiological factors have been proposed to contribute to both migraine and anxiety and depression, including dysregulation of neurotransmitter systems (particularly serotonin, norepinephrine, and gamma-aminobutyric acid), dysfunction in sensory processing and pain modulation, and alteration in brain structure and function (20–22).

In addition, our results demonstrate that depression is linked to increased headache frequency, whereas anxiety is not significantly correlated with headache frequency, duration, or severity. These findings contrast with prior evidence indicating that both anxiety and depression are associated with the frequency and severity of migraine attacks (23–25). The differential associations observed in our study may reflect the distinct pathophysiological roles of anxiety and depression in the pathogenesis of HA-ADD versus primary headache disorders. The relationships between anxiety and depression levels and headache characteristics need further research for confirmation.

This study has several limitations. First, the single-center design and absence of a control group may limit the generalizability of the identified features. Although the female predominance is consistent with the higher prevalence of anxiety and depressive disorders among women in the general population, potential selection bias may further affect the generalizability of our findings. Second, headache characteristics were obtained primarily from patients’ retrospective self-reports of symptoms over the 3 months preceding the initial assessment, which may be subject to recall bias. Finally, analyzing “anxiety and depressive disorder” as a unified entity without subgroup analysis (e.g., by DSM-5 subtypes) limits insights into potential phenotypic variations. Future studies should examine whether different subtypes (e.g., GAD vs. different depressive disorders; DSM-5 codes F41.1, F32–F34) are associated with distinct headache profiles.

In conclusion, this research provides an initial systematic characterization of the clinical profile of patients with HA-ADD, with the core phenotype defined as bilateral, moderate-to-severe dull pain in the temporoparietal regions, associated with phonophobia, restlessness, and agitation. These features differentiate HA-ADD from both migraine and TTH and may facilitate earlier recognition and more precise management of this secondary headache disorder.

## Data Availability

The raw data supporting the conclusions of this article will be made available by the authors, without undue reservation.

## References

[1] Giri S Tronvik EA Hagen K. The bidirectional temporal relationship between headache and affective disorders: longitudinal data from the HUNT studies. J Headache Pain. (2022) 23:14. doi: 10.1186/s10194-022-01388-x, 35062883 PMC8903630

[2] Radat F Milowska D Valade D. Headaches secondary to psychiatric disorders (HSPD): a retrospective study of 87 patients. Headache. (2011) 51:789–95. doi: 10.1111/j.1526-4610.2011.01883.x, 21457254

[3] Lamers F van Oppen P Comijs HC Smit JH Spinhoven P van Balkom AJ . Comorbidity patterns of anxiety and depressive disorders in a large cohort study: the Netherlands study of depression and anxiety (NESDA). J Clin Psychiatry. (2011) 72:341–8. doi: 10.4088/JCP.10m06176blu 21294994

[4] Kasyanov E Yakovleva Y Khobeysh M Gerasimchuk E Mazo G. Lifetime prevalence of recurrent and persistent depression: a scoping review of epidemiological studies. Clin Pract Epidemiol Ment Health. (2025) 21:e17450179372815. doi: 10.2174/0117450179372815250516102324, 40688401 PMC12272091

[5] Huang Y Wang Y Wang H Liu Z Yu X Yan J . Prevalence of mental disorders in China: a cross-sectional epidemiological study. Lancet Psychiatry. (2019) 6:211–24. doi: 10.1016/S2215-0366(18)30511-X, 30792114

[6] Druet-Cabanac A Azzi J Lucchino M Simon V Offredo L Briere JB . Generalized anxiety disorder: epidemiology, burden, and comorbid depression. Curr Med Res Opin. (2025) 41:1053–64. doi: 10.1080/03007995.2025.2529974, 40611531

[7] Houts CR Wirth RJ McGinley JS Gwaltney C Kassel E Snapinn S . Content validity of HIT-6 as a measure of headache impact in people with migraine: a narrative review. Headache. (2020) 60:28–39. doi: 10.1111/head.13701, 31811654 PMC7003926

[8] Kroenke K Spitzer RL Williams JB. The PHQ-9: validity of a brief depression severity measure. J Gen Intern Med. (2001) 16:606–13. doi: 10.1046/j.1525-1497.2001.016009606.x, 11556941 PMC1495268

[9] Toussaint A Hüsing P Gumz A Wingenfeld K Härter M Schramm E . Sensitivity to change and minimal clinically important difference of the 7-item generalized anxiety disorder questionnaire (GAD-7). J Affect Disord. (2020) 265:395–401. doi: 10.1016/j.jad.2020.01.032, 32090765

[10] Puledda F Sacco S Diener HC Ashina M Al-Khazali HM Ashina S . International headache society global practice recommendations for preventive pharmacological treatment of migraine. Cephalalgia. (2024) 44:3331024241269735. doi: 10.1177/03331024241269735 39262214

[11] Sico JJ Antonovich NM Ballard-Hernandez J Buelt AC Grinberg AS Macedo FJ . 2023 U.S. department of veterans affairs and U.S. department of defense clinical practice guideline for the management of headache. Ann Intern Med. (2024) 177:1675–94. doi: 10.7326/ANNALS-24-00551, 39467289

[12] Vandenbroucke JP von Elm E Altman DG Gøtzsche PC Mulrow CD Pocock SJ . Strengthening the reporting of observational studies in epidemiology (STROBE): explanation and elaboration. Epidemiology. (2007) 18:805–35. doi: 10.1017/S0950268808000733 18049195

[13] Olesen J. Some clinical features of the acute migraine attack. An analysis of 750 patients. Headache. (1978) 18:268–71. doi: 10.1111/j.1526-4610.1978.hed1805268.x, 721459

[14] Rasmussen BK Jensen R Olesen J. A population-based analysis of the diagnostic criteria of the international headache society. Cephalalgia. (1991) 11:129–34. doi: 10.1046/j.1468-2982.1991.1103129.x, 1889068

[15] Onan D Younis S Wellsgatnik WD Farham F Andruškevičius S Abashidze A . Debate: differences and similarities between tension-type headache and migraine. J Headache Pain. (2023) 24:92. doi: 10.1186/s10194-023-01614-0, 37474899 PMC10360340

[16] Ran Y Yin Z Lian Y Xu Y Li Y Liu J . Gradually shifting clinical phenomics in migraine spectrum: a cross-sectional, multicenter study of 5438 patients. J Headache Pain. (2022) 23:89. doi: 10.1186/s10194-022-01461-5, 35883029 PMC9327365

[17] Bjørnelv S Lydersen S Mykletun A Holmen T. Changes in BMI-distribution from 1966-69 to 1995-97 in adolescents. The young-HUNT study, Norway. BMC Public Health. (2007) 7:279. doi: 10.1186/1471-2458-7-279, 17916233 PMC2082034

[18] Guruswamy A Swamy S Kurpad KP. Clinical profile of tension type headache in a medical college with special emphasis on triggering factors. Neurol India. (2022) 70:1958–62. doi: 10.4103/0028-3886.359261, 36352594

[19] Haque B Rahman KM Hoque A Hasan AT Chowdhury RN Khan SU . Precipitating and relieving factors of migraine versus tension type headache. BMC Neurol. (2012) 12:82. doi: 10.1186/1471-2377-12-82 22920541 PMC3503560

[20] Viudez-Martínez A Torregrosa AB Navarrete F García-Gutiérrez MS. Understanding the biological relationship between migraine and depression. Biomolecules. (2024) 14:163. doi: 10.3390/biom14020163, 38397400 PMC10886628

[21] Johnson M Filali Y Adegboyo A Eberle M. Mechanistic intersections between migraine and major depressive disorder. J Headache Pain. (2025) 26:157. doi: 10.1186/s10194-025-02097-x, 40634879 PMC12243351

[22] Amaro-Díaz L Montoro CI Fischer-Jbali LR Galvez-Sánchez CM. Chronic pain and emotional stroop: a systematic review. J Clin Med. (2022) 11:3259. doi: 10.3390/jcm11123259, 35743329 PMC9224954

[23] Chu HT Liang CS Lee JT Galvez-Sánchez CM. Associations between depression/anxiety and headache frequency in migraineurs: a cross-sectional study. Headache. (2018) 58:407–15. doi: 10.1111/head.13215, 29044546

[24] Jafari E Kazemizadeh H Togha M Haghighi S Salami Z Shahamati D . The influence of anxiety and depression on headache in adolescent migraineurs: a case–control study. Expert Rev Neurother. (2022) 22:1019–23. doi: 10.1080/14737175.2022.2154657, 36621531

[25] Oh K Cho SJ Chung YK Kim JM Chu MK. Combination of anxiety and depression is associated with an increased headache frequency in migraineurs: a population-based study. BMC Neurol. (2014) 14:238. doi: 10.1186/s12883-014-0238-4, 25494868 PMC4279894

